# Resolving a diagnostic challenge: first PCR-based detection of *Legionella longbeachae* in Italy

**DOI:** 10.1128/spectrum.00860-25

**Published:** 2025-06-25

**Authors:** Silvia Guerriero, Marilena La Sorda, Brunella Posteraro, Flavio De Maio, Sara Cardinali, Pierluigi Del Vecchio, Giovanni Addolorato, Claudia Tarli, Rita Murri, Massimo Fantoni, Maurizio Sanguinetti, Carlo Torti

**Affiliations:** 1 Dipartimento di Sicurezza e Bioetica, Università Cattolica del Sacro Cuore 225080, Rome, Italy; 2 Dipartimento di Scienze di Laboratorio ed Ematologiche, Fondazione Policlinico Universitario A. Gemelli IRCCS 18654, Rome, Italy; 3 Unità Operativa “Medicina di Precisione in Microbiologia Clinica,” Direzione Scientifica, Fondazione Policlinico Universitario A. Gemelli IRCCS 18654, Rome, Italy; 4 Dipartimento di Scienze Mediche e Chirurgiche, Fondazione Policlinico Universitario A. Gemelli IRCCS 18654, Rome, Italy; Indiana University School of Medicine, Indianapolis, Indiana, USA; Mayo Clinic, Rochester, Minnesota, USA

**Keywords:** *Legionella longbeachae*, antigen testing, PCR testing

## LETTER

The diagnosis of *Legionella* pneumonia, primarily a cause of community-acquired pneumonia, traditionally relies on urinary antigen testing, culture, and serology, all of which have limitations in detecting non-*Legionella*. *pneumophila* species (1). *Legionella longbeachae*, an emerging global cause of *Legionella* pneumonia (2), is often underdiagnosed unless molecular assays such as polymerase chain reaction (PCR) are applied (3).

Here, we report the first identification of *L. longbeachae* in our Italian center using a CE-marked real-time PCR assay, the Respiratory Bacterial ELITe MGB Panel assay. This multiplex PCR assay detects *Mycoplasma pneumoniae*, *Chlamydia pneumoniae*, and *L. pneumophila*/*L. longbeachae* DNA in respiratory samples (https://www.elitechgroup.com/molecular-diagnostics-intl/product/respiratory-bacterial-elite-mgb-panel/), adding to established NAAT assays (4–7).

An 81-year-old male with chronic obstructive pulmonary disease complicated by chronic respiratory failure, and a history of arterial hypertension, cerebral vasculopathy, and a previous multifocal pneumonia of unknown etiology, was admitted with fever (38°C), cough, confusion, and acute worsening of respiratory failure. He denied exposure to soil or potting compost. Hyponatremia and diffuse ground-glass opacities with evolving lobar consolidations on high-resolution CT scan raised suspicion of *Legionella* pneumonia.

Serologies for *Chlamydia* and *Mycoplasma* were negative. Urine antigen testing for *L. pneumophila* serogroups 1, 3, 5, 6, and 8 (SD Biosensor STANDARD F *Legionella* Ag FIA test) was negative. However, the SSI Diagnostica A/S ImmuView *L. pneumophila* and *L. longbeachae* urinary antigen test (8) yielded a positive result for *L. longbeachae* and a negative result for *L. pneumophila* serogroup 1. Bacterial cultures from a sputum sample, including those on *Legionella*-selective buffered charcoal yeast extract (BCYE) medium (9), yielded growth of *Klebsiella pneumoniae* but were negative for *Legionella* species. PCR performed on the same sputum sample using the ELITe MGB Panel assay detected *L. pneumophila/L. longbeachae*, whereas the FDA-cleared FilmArray Pneumonia Panel assay, which only detects *L. pneumophila* (4), was negative. Positive controls included *L. pneumophila* ATCC 33823 and *L. longbeachae* ATCC 33462.

**Fig 1:**
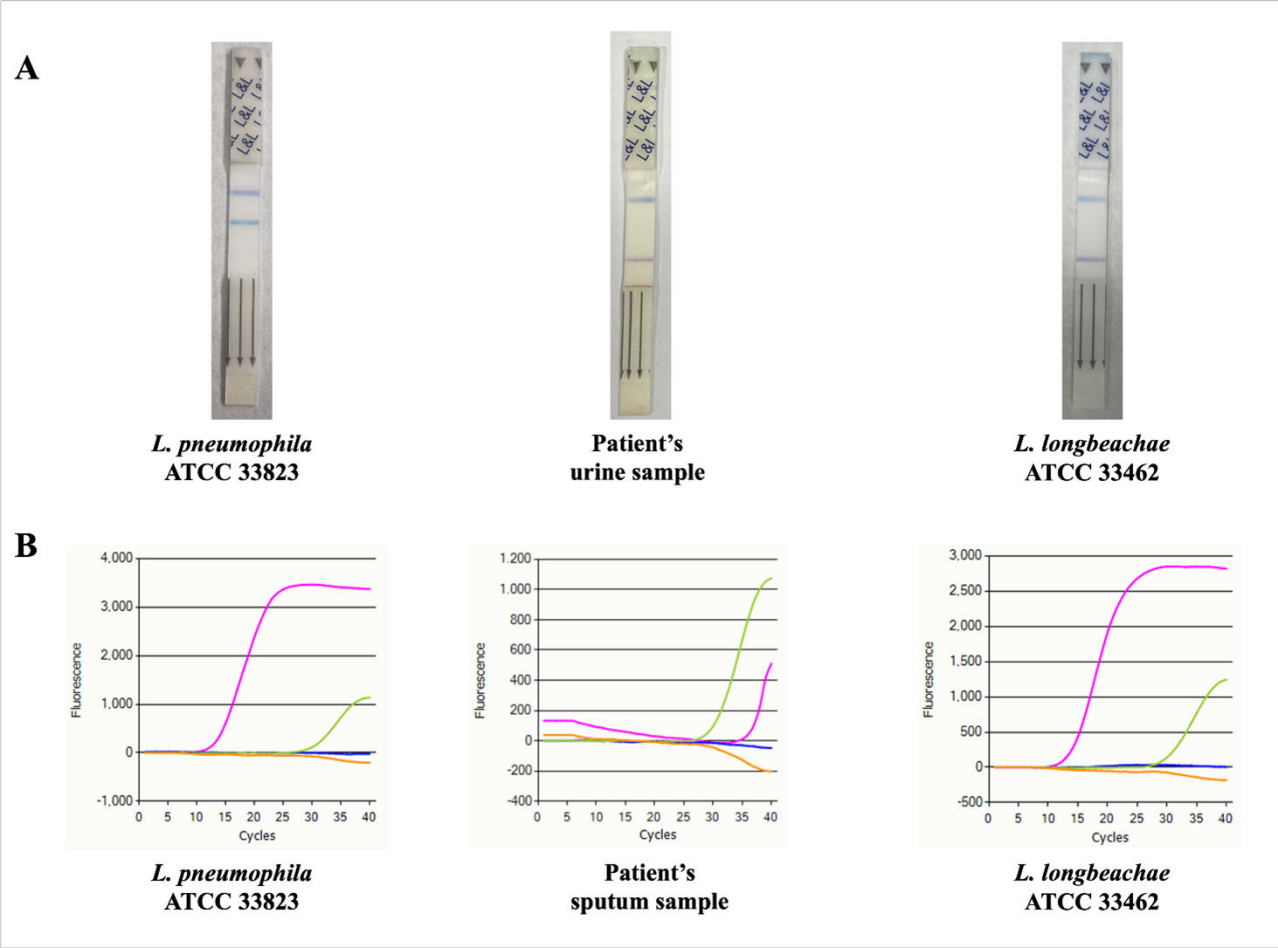
Positive *L. longbeachae* detection results from the pneumonia patient’s samples. Results are shown alongside those obtained in parallel with *L. longbeachae* ATCC 33462 and *L. pneumophila* ATCC 33823 reference strains, which served as positive controls. (**A**) The urine sample was analyzed using the SSI Diagnostica A/S ImmuView *L. pneumophila* and *L. longbeachae* urinary antigen test, yielding a *L. longbeachae*-specific immunochromatographic band. A similar band was visible in the *L. longbeachae* positive control, while no *L. pneumophila*-specific immunochromatographic band—detected in the positive control—was observed. (**B**) The sputum sample was analyzed using the Respiratory Bacterial ELITe MGB Panel assay, yielding a *L. pneumophila*/*L. longbeachae*-positive amplification curve (pink), crossing the fluorescence signal baseline at a threshold cycle (Ct) of 35.6, below the positivity threshold of 40 cycles. The internal control was amplified at a Ct of 28.3 (green), while no amplification was detected for *Chlamydia pneumoniae* (blue) or *Mycoplasma pneumoniae* (orange). Similar positive amplification curves were obtained with both *L. pneumophila* and *L. longbeachae* reference strains, with Ct values of 14.0 and 15.0, respectively (pink).

Based on positive *L. longbeachae* results (illustrated in Fig. 1), intravenous levofloxacin (750 mg daily) was started, a first-line therapy—along with azithromycin—for *Legionella* pneumonia (1). Fluoroquinolones, such as levofloxacin, show higher *in vitro* activity against *L. longbeachae* clinical isolates compared to macrolides (10). Therefore, choosing fluoroquinolones over azithromycin could be advantageous when *L. longbeachae* (rather than *L. pneumophila*) is the causative agent in pneumonia cases. The patient showed progressive clinical and radiological improvement, and antibiotic therapy was discontinued after 9 days.

This case highlights the role of PCR testing, particularly NAAT assays that detect *Legionella* species beyond *L. pneumophila*, as a reliable alternative to BCYE culture when patients with community-acquired pneumonia test negative for *L. pneumophila* urinary antigen. Importantly, urinary antigen tests can remain positive even after pathogen clearance and treatment, while PCR provides higher sensitivity and specificity by directly detecting bacterial DNA, thereby offering a better correlation with active infection. While *L. longbeachae* culture methods require improvement (11), confirmation of PCR results was unlikely in our case due to the relatively high Ct value, suggesting a low bacterial load and reduced probability of culture positivity.

## Supplementary Material

Reviewer comments

## SUPPLEMENTAL MATERIAL

OPEN PEER REVIEW reviewer-comments.pdf An accounting of the reviewer comments and feedback.

ASM does not own the copyrights to Supplemental Material that may be linked to, or accessed through, an article. The authors have granted ASM a non-exclusive, world-wide license to publish the Supplemental Material files. Please contact the corresponding author directly for reuse.
